# Cervical cancer screening programs for female sex workers: a scoping review

**DOI:** 10.3389/fpubh.2023.1226779

**Published:** 2023-09-29

**Authors:** Léa Vimpere, Jana Sami, Emilien Jeannot

**Affiliations:** ^1^Global Studies Institute, Université de Genève, Geneva, Switzerland; ^2^Geneva School of Health Sciences, HES-SO University of Applied Sciences and Arts Western Switzerland, Geneva, Switzerland; ^3^Gynecology Division, Department of Obstetrics and Gynecology, Geneva University Hospitals, Geneva, Switzerland; ^4^Faculty of Medicine, Institute of Global Health, Geneva, Switzerland; ^5^Addiction Medicine, Department of Psychiatry, Lausanne University Hospital and University of Lausanne, Lausanne, Switzerland

**Keywords:** cervical cancer, cervical cancer screening, female sex workers, programs, global health

## Abstract

**Background:**

Cervical cancer (CC) is the fourth most common neoplasia affecting women worldwide. Female sex workers (FSWs) are among those at highest risk of developing and succumbing to CC. Yet, they are often overlooked in CC screening programs and have limited access to CC healthcare globally. The development of CC screening programs for this high-risk target population is necessary to reduce the global burden of this disease and to reach the World Health Organization’s objective of accelerating the elimination of CC.

**Objective:**

This review summarizes findings on CC screening programs for FSWs that have been implemented worldwide, and assesses their effectiveness and sustainability.

**Methods:**

A scoping review was conducted using the Preferred Reporting Items for Systematic reviews and Meta-Analyses extension for Scoping Reviews (PRISMA-ScR). A literature search was performed on PubMed, Swisscovery, and Google Scholar for studies describing and assessing CC screening programs for FSWs. In addition, targeted searching online Non-Governmental and International Organizations websites identified grey literature. A single reviewer screened titles and abstracts, and extracted data from the research findings.

**Results:**

The search identified 13 articles published from 1989 to 2021. All implemented programs successfully reached FSWs and provided them with CC screening during the study period. The most effective and sustainable strategies were the Screen and Treat approach, introducing CC screening into existing STI services in drop-in or outreach clinics, HPV-DNA self-sampling, and integrating sex-workers-specific services in public health facilities. Follow-up was deemed the main challenge in providing and enhancing CC healthcare to FSWs with rates of loss to follow-up ranging from 35 to 60%.

**Conclusion:**

FSWs are often omitted in national CC screening programs. The further development and improvement of CC healthcare, including follow-up systems, for this high-priority target population are imperative.

## 1. Introduction

Cervical cancer (CC) is the fourth most common neoplasia among women (1, 2) and comes second as the most frequent cause of cancer death reported by each country (3). The burden of CC is mainly borne by developing countries in Sub-Saharan Africa, South America, and South-Eastern Asia (3, 4). More than 95% of CC are attributable to human papillomavirus (HPV) infection, which is the most common sexually transmitted infection (STI) (5). HPV16 and 18 are responsible for nearly 50% of high-grade cervical precancers (5). Other risk factors such as smoking, immunodeficiency, use of oral contraceptives, and the number of sex partners have also been associated with an increased likelihood of developing CC (6). Moreover, low socio-economic status and education levels, stigma, clinical exam not being requested by healthcare professionals, lack of accessible screening facilities, and disbelief in public health facilities have been linked with poor knowledge of CC and lack of screening (7). All these factors put female sex workers (FSWs) at high risk of developing and succumbing to CC, hence the higher HPV prevalence among FSWs with respect to the general population (8–11). FSWs are also more affected by high-risk HPV types, which cause most of CC (9, 11–13), with HPV16 being the most common among this population globally (8, 10, 13). Furthermore, compared to the general population, FSWs have a higher prevalence of abnormal Pap smear results (11, 12, 14, 15) and more than twofold increase in the incidence of Cervical Intra-epithelial Neoplasia (CIN) was observed in Kenya (16). Despite regional discrepancies, FSWs show a widespread smoking behavior, a number of sexual partners ranging from two to forty per week, and a prevalent low education level (primary or secondary education) (9, 13, 17).

Effective interventions can prevent the development of and death from CC, including HPV vaccination and CC screening. In 2020, the World Health Organization (WHO) launched a global strategy to accelerate the elimination of CC with the 90–70-90 targets that shall be met by 2030: 90% of girls fully vaccinated with HPV vaccine by the age of 15 years old (y.o.); 70% of women screened by 35 y.o. and again by 45 y.o.; 90% of women with precancer treated, and 90% of women with invasive cancer managed (4). However, HPV vaccination and CC screening programs are mainly available in high-income countries (18) and they do not specifically target under-screened women such as FSWs despite the well-known necessity. Indeed, FSWs have a low or very-low CC screening uptake (19–21) and several factors have been identified as barriers to cervical screening. First, FSWs are considered a highly moving and hard-to-reach population. Second, they often suffer from occupational stigma and poor treatment by healthcare workers. Third, they can also face language and geographical access limitations. Lastly, restricted opening times and provision of services exclusively focused on STI prevention, independently from directly offering CC screening, were identified as limiting factors to accessing CC screening (22, 23). CC knowledge among FSWs highly varies from region to region, and results regarding its influence on screening uptake are contradictory. Yet, tailored educational programs on CC together with highlighting the importance of screening are valuable public health tools.

Only few countries in the world remain without a national CC screening program (24). Despite the acknowledgment that FSWs are at higher risk of developing and succumbing to CC, their limited access to screening emphasizes that specific national CC screening programs have been poorly implemented for this population so far. It is thus necessary to consider them as a high-priority target population to reach the WHO’s objective to accelerate the elimination of CC. Numerous studies have investigated the factors justifying the necessity to implement existing or new CC screening interventions in FSWs. Still, no thorough review identifying CC screening programs for FSWs has been conducted to our knowledge. The aim of this non-exhaustive scoping review is (1) to fill this gap by identifying the existing data about the different CC screening projects and programs for FSWs that have already been implemented worldwide, and (2) to summarize their acceptance, utilization, feasibility, effectiveness, and sustainability. By providing a comprehensive report of the different CC screening strategies, this study will endorse decision-makers with the promoting of strategies reaching the broadest community in order to achieve WHO’s objectives.

## 2. Methods

### 2.1. Literature search

The review was conducted according to the Preferred Reporting Items for Systematic reviews and Meta-Analyses extension for Scoping Reviews (PRISMA-ScR) (25). The research question was framed using the Population, Concept, and Context (PCC) method (26) as following “How CC screening programs for FSWs have been implemented worldwide? Were they adequate/effective in reaching FSWs and were they sustainable?.” Keywords were identified to do a comprehensive literature search between November and December 2022 in the following online databases: Pubmed, Swisscovery, and Google Scholar.

An initial screening of titles and abstracts of the research results was performed by a single reviewer. Inclusion criteria were that the papers should (1) include FSWs in their study population, either address (2) the implementation of a CC screening intervention/program, and/or (3) a CC screening program assessment, and/or (4) FSWs feedback on a CC screening program/intervention. Moreover, articles should be (5) reported in English or French, and (6) have either a qualitative, quantitative, or mixed-methods design. Studies about HPV prevalence and vaccination, comparison between the different HPV testing possibilities, knowledge of CC, and limitations in accessing healthcare and screening services in this specific population were excluded. Duplicates, studies showing an absence of full text or presenting only the protocol, and research not dealing with CC (e.g., STI only, other cancer types or diseases) were also rejected.

Abstracts were selected according to the previous criteria and then underwent a full-text screening. In order to give a comprehensive picture of FSWs dedicated CC screening programs that have been implemented globally, all relevant articles for the topic under investigation and falling within the scope of the review’s research question were included, without date restriction. The reference list of all included studies was reviewed to identify potential additional qualifying papers. Some articles did not meet the inclusion criteria but allowed for the identification of programs that were hand-searched afterward, during the grey literature research process. The detailed search strategy is shown in Table 1.

**Table 1:** Detailed search strategies for study selection.

Data base and date of research	Research equation	Results	Read abstracts	Selected	Exclusion criteria
6.11.22 Pubmed	Sex workers [AND] cervical cancer screening [AND] implementation	6	3	2	Articles about IST/HPV prevalence (2) Articles not about CC screening (2) Total eliminated based on title: 3
6.11.22 Pubmed	Sex workers [AND] cervical screening uptake	11	6	2	Duplicates (3) Articles not about FSWs (2) Article about HPV vaccination in the general population (1) Article not about CC (1) Articles not about CC screening interventions (4) Total eliminated based on title: 5
8.11.22 Pubmed	Primary cervical cancer screening [AND] sex workers	8	4	1	Articles not about FSWs (3) Articles not about CC screening (3) Articles about HPV prevalence (2) Total eliminated based on title: 4
9.11.22 Pubmed	Screen and treat [AND] cervical cancer [AND] sex workers	34	5	2	Duplicates (13) Articles not about FSWs (9) Articles not about CC screening interventions (3) Articles about HPV prevalence (5) Article not about CC (1) Systematic review (1) Total eliminated based on title: 29
11.11.22 Pubmed	Screen and treat [AND] prostitutes [AND] cervical cancer	68	7	1	Duplicates (28) Articles not about FSWs (15) Articles not about CC screening interventions (12) Articles about HPV prevalence (2) Articles not about CC (10) Total eliminated based on title: 61
13.11.22 Pubmed	Human papillomavirus [AND] screening [AND] sex workers	61	5	0	Duplicates (18) Articles not about FSWs (16) Articles not about CC screening interventions (6) Articles about HPV prevalence (15) Articles not about CC (2) Articles about comparison between two tests to detect HPV (4) Total eliminated based on title: 56
16.11.22 Google Scholar (*Filter*: First 10 pages)	Cervical cancer screening programs which worked for sex workers	6,790,000 (100 screened)	37	1	Duplicates (26) Articles not about FSWs (43) Articles not about CC screening interventions (23) Articles about HPV prevalence (2) Article not about CC (1) No full text available (4) Total eliminated based on title: 63
1.12.22 Pubmed (*Filter*: Since 2005)	Sex workers [AND] sexual and reproductive services	337	84	4	Duplicates (30) Articles not about FSWs (137) Articles not about CC screening interventions (19) Articles not about CC (131) Study protocol only (2) Articles about barriers (14) Total eliminated based on title: 253
13.12.22 Pubmed	HPV [OR] human papillomavirus [AND] screening [AND] sex workers	78	5	0	Duplicates (32) Articles not about FSWs (21) Articles about HPV prevalence (18) Articles not about CC (3) Article about HPV vaccination (1) Articles about knowledge regarding HPV (2) No full text available (1) Total eliminated based on title: 73
14.12.22 Swisscovery	Cervical cancer screening programs targeting sex workers	1,095	169	0	Duplicates (135) Articles not about FSWs (348) Articles about HPV prevalence (13) Articles not about CC (510) Articles not about CC screening interventions (17) Articles about HPV vaccination (61) Articles about HPV knowledge (8) Articles about comparison between two tests to detect HPV (1) No full text available (2) Total eliminated based on title: 926

The grey literature was searched in a wide range of online databases through targeted web searching. These databases include, among others, websites from non-governmental organizations (NGOs) and associations whose activities are dedicated to FSWs, the Global Network of Sex Work Projects, Services 4 sex workers, the United Nations (UN) specialized agencies (UNFPA, WHO, UNHCR, UNAIDS), the national health systems and health ministries.

### 2.2. Extracting and charting the results

The studies eligibility was re-verified at the start of the data extraction process. The selected papers were analyzed according to the research objectives. For each article, the following information was extracted: the authors names, the objectives, the design and period, the country where the study was conducted, the sampling method, the sample size, the intervention, the methodology, the main results, the limitations, the ethical consideration, the funding sources, and the conclusion/recommendations. For grey literature, data were retrieved regarding the nature of the intervention/program, feedback from FSWs, and the number of (1) beneficiaries, (2) screening tests performed, (3) treatment provided, and (4) referral, when available.

Once the data were charted, studies involving CC screening programs were grouped. The main results were summarized by the types of screening programs implemented: Screen and Treat, use of existing public health services, diagonal interventions, invitations, well-women outreach clinic, and HPV DNA self-sampling. Studies on programs effectiveness and/or acceptance, sustainability, and FSWs feedback were then synthesized. When studies included data relevant to both aforementioned themes, the information was dissociated and integrated into the most appropriate section. The grey literature findings were reported separately, after the databases’ selected studies results.

## 3. Results

### 3.1. Studies characteristics

**Figure 1:**
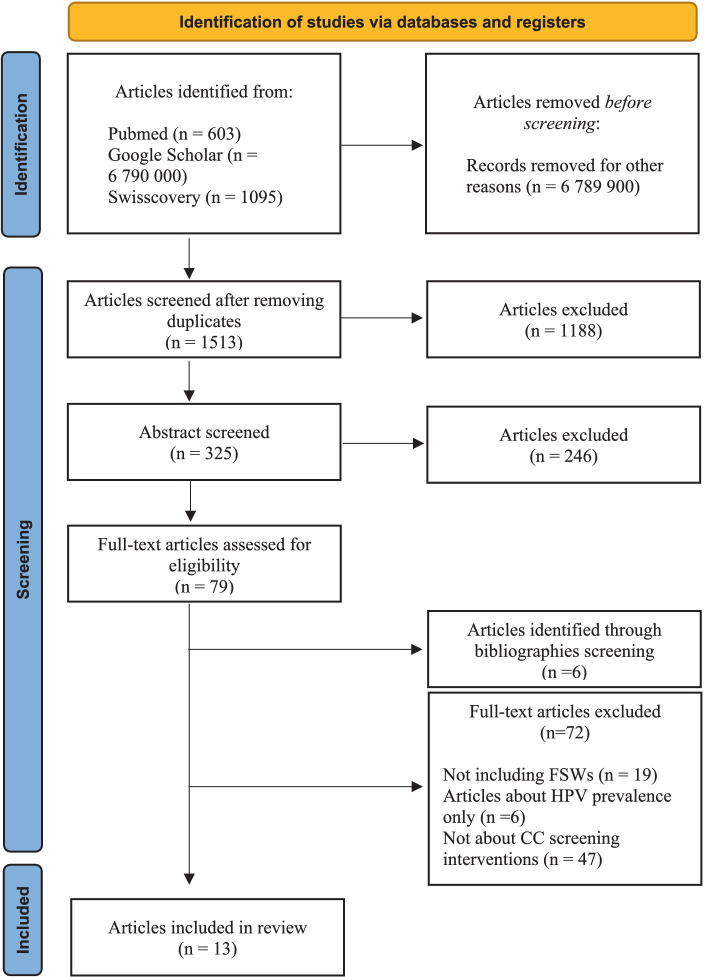
Study selection flow chart. Adapted from: PRISMA 2020 flow diagram for new systematic reviews (27).

Figure 1 shows the selection process for studies included in the review. The literature search yielded a total of 1798 articles. After removing 285 duplicates, 1,513 underwent initial title screening. A total of 1,188 irrelevant publications were excluded leaving 325 articles that underwent abstract screening. From the latter, 79 potentially relevant full-text articles were reviewed, as well as six additional studies, identified through bibliographies screening. In total, 13 research met the inclusion criteria and were included in this review.

Annex 1 summarizes the 13 studies included in this review, published between 1989 and 2021 (28–40). Five of the studies were conducted in Asia, of which two in India (28, 30), two in Hong Kong (31, 32), and one in Bangladesh (36). Four were carried out in Africa, of which two in South Africa (one being the evaluation of the project described in the other) (33, 34), one in Uganda (40), and the last one that addresses a project covering Mozambique, Kenya, and South Africa countries (29). At last, one study took place in Peru (39), and three others in Europe, specifically in the Netherlands (35), Austria (37), and England (38). Among the gathered publications, seven were cross-sectional, four had a mixed-methods design, one was a clinical trial, and one had a qualitative design.

### 3.2. Cervical cancer screening programs

#### 3.2.1. Screen and treat approach

The CC screening programs for FSWs summarized in this review used various approaches. The most prevalent was the Screen and Treat one, with four of the 11 studies about CC screening interventions reporting on it. This method consists in treating the patient immediately after a positive primary screening test (4). In India, the study aimed to evaluate the feasibility of such approach among FSWs. In total, 291 FSWs benefited from this project and it allowed the identification of low (CIN1) and high-grade (CIN2/3) dysplasia among 8% of women (3.4 and 4.8%, respectively). Except one who refused, all of them underwent a same-day cold coagulation treatment of the lesions with no report of side effects after treatment. The high compliance with screening and treatment, indicative of the program’s strong acceptance among FSWs, led the authors to conclude that the approach is feasible for them to access CC healthcare (30).

In South Africa, the Screen and Treat approach was integrated into current HIV care offered by local providers to migrant farm workers and FSWs. Among the 403 women screened, 24% were FSWs, and 27.8% of them had positive Visual Inspection with Acetic Acid (VIA) results, reflecting changes in the cervix cells. Among all VIA-positive participating women, 91.6% underwent cryotherapy, and 4.8% were referred to the hospital for further management. However, the authors did not report disaggregated data about the number of FSWs who received immediate treatment. The program continued after the study period and the analysis of medical records one-year post-implementation revealed that an additional 193 women were screened. Among them, 35.2% underwent treatment and 29% were referred to the hospital. Here again, no disaggregated data were available. Regardless, the authors emphasized the successful integration of a CC screening service into an HIV one. The Screen and Treat approach allows to target and treat hard-to-reach and highly mobile populations such as FSWs. They also highlighted the urgent need to improve infrastructure and referral processes for high-risk populations (33).

Similarly to South Africa, the Ugandan one-year Screen and Treat project for FSWs was integrated into an NGO-led HIV routine care clinic in Kampala. This program reached and screened 719 FSWs, among whom 6% were VIA-positive. From the latter, 65% were referred for a same-day colposcopy in a tertiary hospital, and 35% were lost to follow-up. Of the colposcopic results, 50% of women had low-grade and 26.9% had high-grade cervical lesions, while 15.4% had a suspected invasive cancer. All of them underwent biopsies and 25% had, after all, normal results, 4.1% had CIN1, 54.2% had CIN2/3, and 16.7% had invasive cancer. Most women with biopsy-confirmed precancerous lesions (78.6%) had accepted immediate treatment either by cryotherapy (57.1%) or Loop Electrosurgical Excision Procedure (LEEP) (21.4%). All women with biopsy-confirmed invasive cancer complied with treatment as per the national guidelines. The authors conclusions are comparable to the South African project (40).

In Bangladesh, the Screen and Treat program was evaluated through FSWs interviews about their experiences. The program was seen as an opportunity to have a CC screening and receive free treatment but unlike Indian FSWs, they reported it as painful. In addition, most women feared the medical equipment; some misunderstood the screening procedure and thought they would undergo a hysterectomy. However, the majority felt reassured by the staff, which they described as affectionate, respectful, and trustful (36).

#### 3.2.2. Use of public health services

Two studies reported on the use of existing public health services for FSWs CC screening. In Austria, a mandatory weekly medical examination was enforced in 1873 for FSWs to work lawfully, and a compulsory annual CC screening was introduced in 1988. The Viennese study dates from 1989 and aimed at appraising, among others, the evolution of abnormal Pap smear prevalence in registered FSWs. CC screening was performed through an annual Pap smear on 991 and 958 women in 1988 and 1989, respectively. A decrease in the proportion of FSWs who had abnormal results as well as women who had high-risk lesions was observed. Indeed, in 1988, 8.3% of women had abnormal results and 3.1% of them had a high-risk lesion whereas these proportions dropped to 6.8 and 1.6% in 1989, respectively. The authors did not provide additional data on the management of cervical lesions, limiting the interpretation of the causes of the decrease in cervical lesions and these findings. They only pointed out the considerable difference with the general population figures and the high level of acceptance and compliance among FSWs toward the services provided (37).

In England, GenitoUrinary Medicine (GUM) Clinics are an NHS-free service specialized in sexual and reproductive health (SRH), accessible to anybody (with some of their clinics providing care to specific groups) (41). The authors compared the SRH outcomes and service usage of FSWs with the ones from the other GUM clinics attendees. Overall, 2,704 FSWs visited GUM clinics in 2011, representing 0.4% of patients, and made more visits than other attendees (3.1 visits for FSWs and 1.7 for other women). FSWs were more likely to use non-STI services, such as smear tests, with 12.5% of them who had used the latter against only 1.5% of non-FSWs. Moreover, abnormal smear results were more prevalent in FSWs. Indeed, changes in cervical cells were observed in 32.9% of FSWs and 16.3% of non-FSWs. Interestingly, FSWs mainly visited large clinics providing sex-workers-specific services. It highlights the crucial role of those services in offering broader and targeted sexual health care, such as CC one, to this specific population. The authors concluded that FSWs have access to high-quality SRH services thanks to GUM clinics, but improvements are still to be made (38).

#### 3.2.3. Diagonal interventions

In India, South Africa, Mozambique, and Kenya, the Diagonal Intervention to Fast-Forward Enhanced Reproductive Health (DIFFER) project was implemented to improve SRH services for FSWs. It consisted of both the implementation, in outreach clinics, of numerous SRH interventions designed with and for FSWs through countries baseline needs assessment, and the integration of broader SRH services within existing health facilities (28, 29).

In India, VIA screening and referral for CC were introduced, among other services (e.g., HIV, gender-based violence…), at a sex-worker-led NGO clinic through this program. As a result, the proportion of women screened for CC in 2013 and 2016 increased from 11.5 to 56%. The project allowed for the screening of 1,562 FSWs, of which 46.5% were tested more than once. VIA-reactive results were found in 6.59% of women. Of those, 37.9% were treated with medication and followed up while 62.1% underwent a biopsy. From the latter, 3 FSWs tested positive for biopsy and had a hysterectomy according to the Indian government protocol (28). Although the authors did not provide detailed information about the biopsy results, the treatment these women received strongly suggest they had a CC (28). In Sub-Saharan Africa, the program was significantly effective at improving CC screening only in South Africa. By providing access to screening in 2012, the number of FSWs who had ever been screened doubled in 2015. Moreover, the project successfully increased CC screening uptake in public health facilities. Women were 2.24 times more likely to be tested for CC in a public health facility in 2015 than in 2012. The authors emphasized that it was probably the consequence of the introduction of peer outreach workers in public health facilities. They had the role of being an intermediary between FSWs and healthcare workers and tracking the referred patients (29). They were indeed characterized, by Indian FSWs, as big facilitators for accessing services outside the clinic (28).

#### 3.2.4. Invitations

A program involving direct and indirect invitations for CC screening for marginalized women was tested in the Netherlands. Direct invitations consisted of offering a cervical smear to women attending a medical consultation. Indirect invitations included distributing posters in relevant areas and announcements on a website about the possibility of benefiting from a cervical smear. Additionally to both invitations, mails were sent to known care providers or case managers of the targeted population who could make a cervical smear appointment for their patient. Results were communicated to women through consultations, text messages, and phone calls. In order to prevent patients from being lost to follow-up, a public health safety-net team was responsible for tracking down women who had missed their appointments with the gynecologist or failed to attend a repeat cervical smear after six months. Of the 74 participants, 20 were FSWs but no disaggregated data were available. However, a large majority of women were recruited via the direct invitation approach, and for 83% of women recruited via the indirect one, it was through appointments made by their care providers. In total, 35% of women had positive high-risk HPV test results, and 20% had abnormal smear results. Only women with both positive high-risk HPV test and abnormal smear results were considered screen-positive and referred to a gynecologist (16%). Importantly, the safety-net team has been invaluable in ensuring that all concerned women went to their referral or follow-up appointment. The authors concluded that a proactive individual invitation was crucial to increasing marginalized women’s CC screening as they are omitted by national CC screening programs (35).

#### 3.2.5. Outreach well-women clinic

An outreach clinic providing health services, including Pap smears, was settled at a sex workers dedicated NGO in Hong Kong. Among the 245 FSWs who visited the clinic, 64.5% had never been screened for CC. The program allowed 236 FSWs to undergo such screening, from which 2.5% had reactive changes in squamous cells, 6.8% had CIN 1, and 3% had CIN 2–3. Only 10% of women with abnormal results had had a previous Pap smear in their life. Follow-up was challenging as 11.9% of screened women did not return to the clinic for their results. Moreover, 44.8% of women with abnormal Pap smear results could not be contacted. It concerned only women with reactive changes and CIN1. Of the 55.2% who could be contacted, 24.1% had a repeated cervical smear, and 31.1% were referred for further management. An outreach well-women clinic was deemed as feasible and valuable in preventing CC in FSWs by the authors (31).

#### 3.2.6. HPV DNA self-sampling

In Hong Kong, an HPV DNA self-sampling program was implemented to explore the acceptability and reliability of this screening procedure among FSWs. Participants were first performing the self-collection, then were interviewed before undergoing a Pap test and a clinician collected HPV DNA testing. A total of 76.2% of the 68 participating FSWs had already had a Pap test before the study. Among them, 58.3% stated a preference to use the self-sampling method for future screening, either at home or at a clinic, contrary to 86.7% of women with no history of Pap test. Self-sampling was characterized as more convenient, simpler, less frightening, embarrassing, worrisome, and unpleasant. Only 34.4% were more confident in and preferred clinician sampling (32).

### 3.3. Programs assessment: effectiveness, feedback, and sustainability

The highlighted plus-value of the Screen and Treat approach is that it offers FSWs access to early screening, detection, and treatment of cervical abnormalities with limiting loss to follow-up, which constitutes a major issue in this highly-moving population (30, 40). Nevertheless, the South African project disclosed high rates of loss to follow-up with more than 50% of women who did not present themselves to the one-year post-initial screening check-up. Also, women with suspected invasive carcinoma were referred to the hospital but did not undergo further investigations and were often lost to follow-up as well (33). However, due to the unavailability of data about FSWs and the high mobility of migrant workers, the interpretation of the results from this program is limited. The authors assessed the quality and sustainability of the program 18 months post-implementation. They highlighted the positive impacts on women’s CC knowledge and awareness, understanding of the importance of screening, and access to CC health services. The program was well-accepted by women who gave positive feedback. For its improvement, they recommended extending it to other clinics to increase accessibility and foster screening. They also requested more education as they sought to increase their knowledge about CC, its causes, symptoms, stages, and available treatment options (34). On the other hand, due to high rates of loss to follow-up, program sustainability was challenging to assess. The authors concluded that the program’s persistence for an extended period now reflected that it was, at least, partly sustainable (34). Interestingly, one of the selected studies investigated the barriers to follow-up after abnormal Pap smears among Peruvian FSWs. The results of this study complete the ones from the aforementioned South African program and give insights to better understand high rates of loss to follow-up. Not having been informed of abnormal results, lack of knowledge about CC, its causes and screening possibilities, knowing someone who had a negative experience with abnormal Pap smear follow-up, and being a migrant worker were all factors preventing a follow-up. On the other hand, knowing someone who died of CC, having extensive knowledge of HPV and CC, and social support from family, friends, or partner were all associated with successful follow-up. Overall, the decision to whether pursue or not follow-up care was attributed to fear. A major challenge raised by both FSWs who received and those who did not receive follow-up care was the necessity to miss work days to be treated and recover (39). This is reflected in the study conducted among Bangladeshis FSWs who benefited from a Screen and Treat program, where most of them had difficulties in following the post-treatment guidelines (i.e., abstaining from sex or using condoms consistently for 4 weeks) and 87.5% re-engaged in sex work within a few days following treatment. Among them, half were able to use or negotiate condoms use with clients for all or part of the post-treatment phase. Financial reasons were found to be the main constraint for guidelines non-compliance (36).

Regarding the HPV DNA self-sampling program, FSWs demonstrated a high acceptance of the method and were predominantly positive in using it for future screenings. Moreover, the authors concluded it was a feasible and easy approach to improve access and compliance with CC screening among FSWs. They also stated that access could be improved by proposing HPV DNA self-sampling at NGOs working with and for FSWs (32). FSWs feedback on the Indian DIFFER project was positive, and various benefits were identified. It increased their knowledge of CC, access to screening and treatment, and commitment to regular screening. Interviews conducted with community leaders and partners highlighted the effectiveness and successful implementation of the program. They declared that broader SRH services, such as early CC screening, treatment, and follow-up, can be provided to FSWs and that routine screening is possible (28). Lastly, only one study broached the subject of the necessity to accommodate clinics opening hours to meet FSWs needs and improve intervention effectiveness (31).

### 3.4. Grey literature search results

The extensive review of grey literature allowed for the identification of several programs targeting FSWs and aiming at increasing their access to CC screening.

In Nicaragua, a voucher approach has been implemented to enhance FSWs CC screening. Vouchers were distributed to FSWs who presented them to the private clinics contracted by the projects and served as payment for a Pap smear. Then, the clinics returned the vouchers, the patient’s medical records, and her cytology to the voucher agency. The latter paid the clinics the amount previously agreed for the screening test. If the results were positive, and depending on them, women were given a second voucher for either a 6-month follow-up Pap smear or colposcopy. Ultimately, the agency reported the program outputs and outcomes to the government or the donor agency. During this program, 88% of distributed vouchers were used allowing for the screening of 328 FSWs. Among those, 11.89% had CIN1 and 1.83% had CIN 2/3. All women with positive results were treated. The program successfully reached its objectives: improve coverage, screening, and follow-up of CC in high-risk populations (42). This scheme allowed, among others, for more equity in access to health services and increased service use by vulnerable groups (42). Using this approach has been demonstrated as cost-effective and efficient in delivering CC screening to high-risk women. However, it needs to be adopted by central government and enforced on a large scale to reach the highest number of vulnerable women (42). From 1996 to 1999, such program was already implemented in Nicaragua, offering access to numerous SRH services including CC screening for FSWs. However, no disaggregated data about the latter were made available. Nonetheless, at that time, the program received positive feedback, especially regarding reduced discrimination. The possibility of choice between the different collaborating health centers was also highly appreciated as it removed the geographical limitations to screening access (43).

In 2018, the Kenyan NGO “International Centre for Reproductive Health-Kenya” (ICRHK), in collaboration with the United Nations Population Fund (UNFPA), established a comprehensive SRH delivery program for FSWs (44). Providing CC screening was part of their broader objective to extend access to healthcare services and promote SRH for FSWs. The program provided VIA for CC screening and ensured referral for treatment of advanced lesions. They proposed those clinical services through both drop-in centers and outreach approaches. The latter allowed to extend access to CC screening by considering and reaching FSWs that could not travel to or were hesitant to attend the drop-in clinics. The program also organized events dedicated to FSWs and took that opportunity to raise awareness or provide the clinical services usually offered at the drop-in clinics. Even if there is no specific disaggregated data about the acceptability and increased uptake of CC services by FSWs, the program was successful at involving about 7,500 of them between 2018 and 2021. Moreover, there was a noticeable rise in clinical services utilization, which integrated CC screening, over the project duration. Last but not least, the program received positive feedback from FSWs regarding its beneficial impact on their health (44). Still in Kenya, the CIHEB-Kenya’s CONNECT program is involved in improving CC screening and treatment for FSWs. It provides healthcare workers with training for CC screening and management of precancerous lesions in 13 drop-in clinics (45).

The North Star Alliance delivers healthcare to mobile populations across 10 African countries in shipping containers converted into clinics. Most of these are tailored to the needs of the targeted population as they are open late, enhancing and allowing access to healthcare services beyond conventional working hours (46). Sex workers accounted for 34% of their clients in 2019 and this proportion has increased yearly since. One of their clinics provides CC screening and treatment (47–49). The Cameroon Baptist Convention Health Services aimed at preventing CC among FSWs. They implemented the Women’s Health program in collaboration with a FSWs dedicated NGO in 2020. It allowed for the screening of almost 800 FSWs and the treatment of more than half of the women with positive results in three large Cameroonian cities (50). In South Africa, the Lifeline NGO is very active in improving FSWs access to healthcare services. In 2022, 39 Pap smears were performed on FSWs in a newly operationalized Mobile Unit (51). The Sex Workers Education and Advocacy Task Force (SWEAT), supported by the UNFPA, conducted as part of their integrated model for sex work programming a specific one-month Pap smear campaign in a South-African sub-district. The peer education team provided education both on CC and screening, and referred around 50 FSWs for a screening Pap smear test (52). The organization wished their model served as a framework to expand FSWs services; however, there is a lack of information regarding the pursuit of the Pap smear campaign over time. In Cambodia, the Women’s health program implemented at the Mercy Medical Center offers HPV and CC education, direct screening to FSWs, and a clinician VIA training program. They work closely with FSWs dedicated NGO and referral partners to reduce CC morbidity and mortality in this high-risk group (53, 54). Regrettably, no additional data beyond the aforementioned is available from the various identified programs.

Other projects dedicated to FSWs and providing CC screening were identified in the United States of America (55, 56), the United Kingdom (57–61), Denmark (62), Belgium (63), Mozambique (64), Kenya (65, 66), South Africa (67), and Ethiopia (68). Yet, neither data nor disaggregated data were available regarding screening uptake, follow-up, and effectiveness of those services.

## 4. Discussion

Cervical cancer is a major global health problem disproportionately affecting FSWs worldwide. The WHO included CC prevention, assessment, and treatment as essential health interventions in their recommended package for sex workers (69). Yet, they still encounter difficulties in accessing health care services, including CC screening and treatment of cervical dysplasia. The use of an outreach strategy is widespread in order to increase vulnerable populations health screening compliance (70). Different types of outreach methods exist and the literature has shown that accessing outreach services was correlated with a more regular cervical screening among FSWs (22). The projects identified in this paper also highlight the various complementary possibilities to reach FSWs for CC care and hence reduce their morbidity and mortality related to this disease.

There is little research exploring the implementation of FSWs’ targeted CC screening approaches and where evidence exists, it is largely limited to reports of service utilization rather than intervention effectiveness. The available literature mainly focuses on STI screening and treatment for this population. This review emphasizes the need to extend those services to improve FSWs health and access to health services. Some successful programs summarized in this paper (28, 33, 40) showed the feasibility and acceptability of incorporating CC screening into STI services offered to FSWs. Such programs also allow to reach and provide CC screening to target women (i.e., HIV-positive ones) as their risk of developing CC is six-fold higher (71). Moreover, offering CC screening and treatment increases the scope of services provided to FSWs and responds to their expressed needs to access such services. For instance, in a research about HIV-services preferences among FSWs in Malawi, CC screening was the preferred additional service to pre-exposure prophylaxis service delivery (72). In another study, FSWs exposed their preoccupations regarding diseases other than HIV, such as CC, and sought to access CC screening services (73). Lastly, in an HPV vaccination trial, FSWs claimed their primary motivation to participate was to receive cervical screening (74). In conclusion, the broader SRH needs of FSWs are often overlooked. The expansion of clinical services beyond STIs and HIV care is necessary, and it includes expanding the availability of CC screening services (75).

In the identified programs, the VIA method was predominantly used for screening. Due to quality assurance challenges, VIA is no longer recommended as a primary screening test by the WHO, which urges programs using VIA to shift for HPV DNA testing, either through clinician or self-collected samples (4). Results from previous studies demonstrated that HPV-DNA self-sampling increases participation in CC screening among under-screened and hard-to-reach populations (76, 77). The success of the HPV DNA self-sampling program among Hongkongers FSWs reinforces these findings (32). Yet, this was the only identified research involving this screening method among FSWs. Previous research has demonstrated a strong acceptance for HIV self-testing (78), the latter could be distributed together with HPV-DNA self-sampling. However, despite promising results (32), more research is needed to determine whether or not HPV DNA self-sampling is effective in increasing CC screening uptake in this specific population. Moreover, with this method, follow-up remains a major challenge (79), especially among underprivileged women (80), as discussed later in details. An ongoing project in Ivory Coast aims at developing, documenting, and analyzing a comprehensive sexual and reproductive healthcare package among FSWs by providing those services using both mobile and fixed clinics. One of its main objectives is to evaluate the impact of HPV infections and associated cervical lesions as well as the added value of HPV PCR for CC primary screening (81). In India, the PREvention and SCReening Innovation Project Toward Elimination of Cervical Cancer (PRESCRIP-TEC) started in 2022 with the objective of approaching FSWs. The aim is to apply the WHO’s protocol for CC screening in under-screened populations by implementing high-risk HPV self-testing as the primary screening test. They will evaluate the changes in coverage and uptake of CC screening, as well as adherence to follow-up and treatment recommendations after screening (82). The outcomes of these programs along with an assessment of the strategies implemented to enhance follow-up, particularly after positive test results, will be crucial and have the potential to serve as a framework for future CC screening programs for FSWs.

Overall, follow-up has been stated as one of the main challenges in numerous programs (31, 33, 35, 40). Several factors have been determined as barriers to follow-up (39), and the transient nature of FSWs (75) is one of them. The Screen and Treat approach was often used to overcome the latter as women are screened and treated on the same day. Despite being effective in providing access to CC healthcare, well accepted and seen as valuable by FSWs (30, 34, 36, 40), following the post-treatment guidelines was challenging and seemed nearly impossible as FSWs depend on their activity to meet their needs (36). Moreover, the studies still reported high rates of loss to follow-up. This was observed either for referred women who needed further investigation (33, 40) or for the ones who needed a one-year follow-up visit (33) following the WHO’s recommendation (4). The high rates of loss to follow-up among this population are not only observed in CC care (83), and addressing barriers is crucial to reduce FSWs CC morbidity and mortality. Some programs (28, 29, 35) have implemented effective approaches to mitigate the loss to follow-up among referred women. These strategies involved establishing a team, sometimes composed of peer outreach workers (28, 29), responsible for tracking women and ensuring they attended their appointment by sometimes recalling them (35). Recall efforts, mainly through phone calls or texting, effectively improved attendance rates for one-year follow-up visits among HPV-positive women in the general population (84). However, while receiving health tips and appointment reminders via texting was part of FSWs preferred services for CC screening and treatment (85), the effectiveness of recall methods in reducing loss to follow-up in this population remains undocumented. As such, further research is needed to implement these methods and tailor them to FSWs specific needs. Mitigating loss to follow-up can also be accomplished by addressing other identified restrictions. In Peru, the study results led to the implementation of two FSWs dedicated interventions in the clinic. First, a standardized form for recording Pap smear exam dates, results, and follow-up care was created to ensure women were informed of their results. Second, an educational brochure was produced and distributed to FSWs during their visits to increase their HPV and CC knowledge (39). There is an evident need to develop robust follow-up systems adapted to FSWs to improve programs effectiveness and sustainability. However, further research is necessary to assess the most effective way, considering financial and human resources limitations.

Regarding sustainability, the continuation of most of the identified interventions following the completion of the study remains unclear. Therefore, the long-term effectiveness of these projects for improving FSWs access to CC screening and treatment is unknown. Three studies (28, 34, 36) address the post-project phase however, only one project was certainly ongoing following its implementation (34). One research (28) evaluated the implemented project but its persistence is ambiguous, and a second one (36) clearly stated the program was stopped due to funding limitations. An alternative is the integration of sex-workers-specific services into public health facilities. As previously discussed, introducing health navigators in the public health facilities and giving sensitization training to healthcare workers resulted in increased use of public health services for CC screening (29, 38). This alternative directly responds to FSWs expressed needs for friendly, non-stigmatizing, non-judgmental services and FSWs-targeted interventions (85). Moreover, these results are consistent with previous findings where positive experiences within healthcare services and sex-work-specific services improve FSWs engagement with healthcare and health outcomes (86). Nonetheless, it entails working on destigmatizing and decriminalizing sex work.

Sex work legislation highly varies across countries, and only New Zealand and two Australian states have fully decriminalized sex work (87). Some countries adopted a regulatory model and have enforced mandatory health screening for FSWs to work legally (87, 88). This was the model analyzed in the study conducted in Vienna (37), and despite being published in former times, it is still currently used (89). However, mandatory health screening, besides reinforcing stigmatization (90), is considered a fundamental human rights abuse (91) and must cease (92). Thus, this method should not be considered to improve FSWs access to CC screening and treatment. Evidence has shown that criminalization of sex work has negative consequences on sex workers, who experience further marginalization, poorer physical and mental health outcomes, and access to health services (88, 93). Furthermore, criminalization largely reduces FSWs access to SRH services (94). In order to remove the barriers to essential health services such as CC screening and treatment resulting from prohibitionist policies, the WHO recommends that all countries work toward sex work decriminalization (69). Empirical evidence has demonstrated that health promotion programs were more developed and used by FSWs in decriminalized environments. Moreover, greater health education, access to health services, and health-seeking behaviors were observed (93). Decriminalization also promotes less stigmatization and, consequently, could enhance access to public healthcare facilities for CC screening.

As highlighted in this review, NGOs invaluable work make them essential to respond to the needs of FSWs, and not solely regarding CC. Nonetheless, there is a paucity of data stemming from their implemented programs limiting their evaluation, interpretation, and utilization hence the necessity of improving NGOs data collection. As main actors in the field of sex work, they can improve and enhance sex work research through good data collection and reporting. Moreover, their reliance on external funding (95) burdens their programs sustainability with potential considerable consequences on FSWs health. Despite the previously mentioned programs, various ongoing ones have been identified during the search. The results of these projects will be essential for research and future global health strategies for CC. The South African WITS Reproductive Health and HIV Institute launched a key population program for sex workers and transgender individuals in 2018, ending in 2023. One of its main objectives is to increase access to Pap smear using a targeted peer outreach approach, tailored services, and sensitization of community partners to reach most women and increase CC screening uptake (96). In Europe, the ongoing CBIG-SCREEN project launched in 2020 aims to tackle inequality in CC screening, by improving access to and provision of CC screening to vulnerable women, including sex workers (97, 98). Last but not least, South Africa launched in 2019 its new National sex workers plan in which access to annual Pap smear for CC screening is part of their objectives (99). Once more, the outcomes of this groundbreaking initiative could inform the development of future national plans.

Finally, HPV infection with high-risk types is preventable through vaccination, thus decreasing the risk of developing cervical cancer. The literature has identified pros and cons regarding FSWs vaccination against HPV. Factors in favor of offering them HPV vaccination include the fact that the vaccine immunogenicity is very good, even in women previously exposed to HPV types contained in the vaccine. Second, it lowers the recurrence of HPV-caused disease after a primary surgical treatment. Third, it prevents persistent infection and first-stage CIN. Last but not least, vaccination can protect FSWs against HPV types they have not been infected with (100). However, despite those identified factors evidence is still lacking and further research is needed to inform decision-making (100). Moreover, there is a persistent global disparity in HPV vaccine accessibility (101) with limited access to free-of-charge vaccines for certain populations. The HPV vaccination cost is a major determinant influencing vaccination intentions in the general population (102), and in FSWs (103, 104), although most of them are willing to be vaccinated (105). Consequently, only few HPV vaccination programs for FSWs have been identified in the literature. In India, a project that started in 2022, consists of administrating the first dose of an HPV vaccine to eligible women undergoing a Pap smear (106). Assessing the acceptance and effectiveness of such program is essential to support the development of other vaccination strategies for FSWs. Lastly, considering that the expansion of girls HPV vaccination plays a crucial role in decreasing CC mortality (107), these efforts may impact FSWs risks of HPV infection and development of cervical lesions.

### 4.1. Strengths and limitations

This review provides an overview of CC screening programs for FSWs, using both peer-reviewed publications and grey literature. It highlights successful strategies and provides insights for policymakers to improve FSWs CC healthcare. However, results should be interpreted with caution due to some limitations that need to be addressed. First, the chosen research equations and keywords may have led to the exclusion of literature. Indeed, literature with “vulnerable” or “marginalized” women as a population without further specifications was not included in the analysis, even though FSWs may have been encompassed. Second, since screening for eligibility and data extraction was performed by only one researcher, some relevant studies may have been omitted. Third, all reviewed articles are published in English which may exclude pertinent literature published in other languages. Fourth, given that sex work legislation, types of sex work, and FSWs characteristics vary significantly globally, the generalizability of the identified programs’ results to other settings might be limited. Fifth, the absence of disaggregated data in studies including other populations than FSWs narrows the conclusion that can be inferred from the programs. Lastly, the quality of the selected studies was not assessed as it is not the objective of a scoping review.

## 5. Conclusion

FSWs are at higher risk of developing and succumbing to CC. Yet, they have a limited access to CC screening that results in very-low screening uptake. This review presents various possibilities to foster CC screening and treatment among FSWs in the aim of meeting WHO’s objective to reduce CC morbidity and mortality by 2030. Overall, adapting current national CC screening programs proved to be necessary as they require including FSWs. Moreover, the literature has demonstrated the effectiveness of strategies such as the Screen and Treat, drop-in and outreach clinics providing both CC and STI services, along with HPV-DNA self-sampling, which has demonstrated a significant potential in improving FSWs CC healthcare. The introduction of sex-workers-specific services in public health facilities is also an effective and sustainable approach. However, it calls for substantial efforts to destigmatize but more importantly, to decriminalize sex work. This study also highlights the necessity of refining the existing follow-up systems, both before and after (1-year follow-up) treatment, to ensure access to care for women with cervical lesions. Furthermore, it is fundamental to support and strengthen NGOs work so that to provide FSWs with CC healthcare. Finally, it is necessary to continue implementing effective education campaigns on HPV and CC, with an emphasis on the importance of screening for target populations.

## Data availability statement

The original contributions presented in the study are included in the article/Supplementary material, further inquiries can be directed to the corresponding author.

## Author contributions

LV and EJ had the original idea for the review. LV collected and analyzed the data and drafted the manuscript. EJ reviewed and provided feedback on all drafts. JS reviewed and provided feedback on the last two versions of the manuscript. All authors contributed to the article and approved the submitted version.

## Abbreviations

CC: Cervical cancer
CIN: Cervical Intra-epithelial Neoplasia
FSW: Female sex worker
GUM: GenitoUrinary Medicine
LEEP: Loop Electrosurgical Excision Procedure
NGO: Non-Governmental Organization
STI: Sexually Transmitted Infection
VIA: Visual Inspection with Acetic Acid
