# Supplementary material for: Cervical cancer screening programs for female sex workers: a scoping review
Source: Front Public Health. 2023 Sep 29;11:1226779. doi: 10.3389/fpubh.2023.1226779 (PMC10570451; doi:10.3389/fpubh.2023.1226779)
Supplement: Supplementary file 1 [file Table_1.DOCX]

Supplementary Material

Cervical cancer screening programs for female sex workers: A scoping review

Léa Vimpere, Jana Sami, Emilien Jeannot*

*** Correspondence:** Emilien Jeannot: Emilien.Jeannot@unige.ch

# Supplementary Figures and Tables

**1.1 Supplementary Table**

| ***« Delivering community-led integrated HIV and sexual and reproductive health services for sex workers: A mixed methods evaluation of the DIFFER study in Mysore, South India » (1)*** Sushena Reza-Paul, Lisa Lazarus, Raviprakash Maiya, K. T. Venukumar, Bhagya Lakshmi, Anuradha Roy, Partha Haldar, Michele Andina, Yves Lafort, Robert Lorway (2019) | | | | | |
| --- | --- | --- | --- | --- | --- |
| Study objective  Study design  Country/Year of study | Sampling  Intervention | Methodology | Main results (regarding cervical screening only) | Limitations  Ethical consideration  Funding sources | Conclusion/ Recommendations |
| **Objective**: To evaluate the uptake rates for SRH services among FSWs after the implementation of the DIFFER program.  **Design**: Mixed-methods study  **Country**: India (Mysore) / 2013-2016 | **Sampling**:  Respondent-driven sampling (selection of 8 “seeds” from various sex work networks, then each seed invited 5 FSWs through coupons distribution and so on)  *Cross-sectional*:  Base-line: n=458  End-of-project : n=415  *Focus group discussions*: Purposive sampling (n=8 to 10)  *Key Informant interviews*: Purposive sampling (n=9)  **Intervention**:  • Strengthening (1) existing community mobilization & peer outreach, and (2) HIV/STI services offered at the Ashodaya clinic  • Introducing long-acting family planning methods + increasing counseling at the Ashodaya clinic  • Referrals and linkages with government hospitals by healthcare navigators  • Preventing sexual and gender-based violence  • Initiating a “Well Women Clinic” at a hospital for people living with HIV to integrate SRH services for HIV-positive women  • Introducing free of cost VIA screening and referral for CC at the Ashodaya clinic (trainings were received by the existing staff)  The project was implemented by a sex worker-led organization (Ashodaya) | - Cross-sectional surveys**:** Base-line and end-of-project face-to-face interviews using paper-based questionnaires  “The quantitative outcome indicators that were examined […] were the use of HIV/SRH commodities and services by sex workers that include condom use, STI care, HIV testing and care, use of contraception, and cervical cancer screening and care.”(1)  “Changes in the uptake of SRH services between the two surveys were assessed for statistical significance by merging the baseline and end-of-project datasets and fitting a logistic regression model” (1)  - **Focus group discussions**: Base-line (n=6) and end-of-project (n=8), audio recorded and English transcribed discussions. Transcripts were coded for key themes and emergent categories and then analyzed by thematic and content.  The guide focused on :  • Knowledge and use of SRH services  • Access to SRH services  • Stigma and discrimination faced  • Outreach  • Community mobilization  • Satisfaction with the DIFFER services (for end-of-project discussions only)  - **Key informant interviews**: Base-line and end-of-project interviews of community leaders and partners, by the Ashodaya staff and researchers. Either face-to-face with audio recording and English transcription or via phone with extensive notetaking during and immediately after the interview. Then those notes were coded for emergent themes.  Focused on :  • Feasibility & sustainability & appropriateness of the interventions  All the data collected “were triangulated with the services statistics obtained from the Ashodaya clinic and Well Women Clinic for the intervention duration (2013 to 2016).” (1) | **Surveys:**  - Major increase of the number of women who had been screened for CC between the 2 surveys (from 11.5% to 56%) (AOR = 9.85, p< 0.001)  - 2302 VIA tests were conducted at the clinic (1562 FSW were tested, 836 (53.5%) tested once, and 726 (46.5%) tested more than once).  - 6.6% of tested FSW had a reactive VIA, and among them:  • 37.9% were treated and  followed up and  symptoms were resolved  with medication  • 62.1% underwent biopsy  • 4.7% (3 FSW) tested  positive for biopsy and had  a hysterectomy  **Focus group**:  - New SRH services increased:  • Knowledge of CC and  access to testing among the  focus group participants  • Women’s own commitment  to regular screening  - FSW were able to access screening and effective treatment for their CC  - Health care navigators and referrals were big facilitators of services access outside the Ashodaya clinic for FSWs  **Key informant interviews**:  - The project has shown that:  • All needed services (beyond  HIV services) can be  provided, including early  CC screening, follow-up  and early treatment, to  FSWs.  • Routine screening with  trained staff is possible  and necessary | - Data were adjusted for RDS effect but no description/details of the other characteristics (potential cofounders).  - RDS sampling lead to risk of selection bias  - Lack of control group  - Base-line and end-line cross sectional surveys did not necessarily include the same FSWs  - Risk of reporting (social desirability) and classification bias  **Ethical consideration**:  - Written informed consent given by all study participants  - Ethics obtained from Institutional Ethics Committee  **Funding**: From the European Union Seventh Framework Programme + KSAPS (Karnataka State AIDS Prevention Society) | - Including CC screening in SHR services for FSWs allowed the early detection, treatment, and referral when needed, of women with cervical abnormalities  - “SRH service uptake, [including screening counseling and treatment], can occur in conjunction with HIV services offered to sex workers” (1)  **-** The project was effective in scaling up access to CC screening and treatment  - Identification of service gaps to move towards comprehensive service delivery was an effective approach  - There is a need to not solely focus on STIs and HIV prevention to improve health and access to health services of FSWs  - There is a need that governments and NGO’s move toward a more integrative HIV/SHR model, and such project help in the recognition of the effectiveness of comprehensive SHR package and protocols. |

| ***“Effect of a ‘diagonal’ intervention on uptake of HIV and reproductive health services by female sex workers in three sub-Saharan African cities” (2).*** Yves Lafort, Letitia Greener, Faustino Lessitala, Sophie Chabeda, Ross Greener, Mags Beksinska, Peter Gichangi, Sally Griffin, Jenni A. Smit, Matthew Chersich, and Wim Delva (2018) | | | | | |
| --- | --- | --- | --- | --- | --- |
| Study objective  Study design  Country/Year of study | Sampling  Intervention | Methodology | Main results (regarding cervical screening only) | Limitation  Ethical consideration  Funding sources | Conclusion/ Recommendations |
| **Objective**: To test a diagonal approach (⇔ ”Delivery of targeted clinical services (vertical) with improving access to the general health services (horizontal) and establishing linkage between them” (2)) to improve uptake of SRH services by FSWs  **Design**: Mixed-methods  **Country**: South Africa (Durban), Mozambique (Tete), Kenya (Mombasa) / 2012-2015 | **Sampling** :  Respondent-driven sampling (identification of “seeds” with the help of peer educators, then each seed invited 3 FSW through coupon distribution and so on)  *Durban*: n= 400  *Tete*: Base-line n=311, end-line n=404  *Mombasa*: Base-line n=400, end-line n=403  **Interventions**:  • Facilitating access to general health facilities  • Targeted peer outreach  • Targeted clinical services  • FSW empowerment | **Mixed methods :**   - Measurement of quantitative indicators of service uptake - Focus-group discussions and semi-structured key informant interviews   **Cross sectional surveys :** Base-line and end-line face to face interviews using a questionnaire assessing the need for contraception, STI care, HIV testing and care, CC screening (for women 30 years or older) and sexual violence services. Then if FSW had a need, assessment of the use (or not) and location of services used.  “Changes in uptake of SRH services between the two surveys were assessed for statistical significance by merging the baseline and end-line datasets, and fitting a logistic regression model” (2) | - Increased in the number of women who had ever been screened for CC from 31.8% to 60% in Durban (p = 0.001)  - Increased of care seeking at public health facilities for CC screening: Women interviewed at end-line had 2.24 more odds of being tested for CC in a public health facility than FSWs interviewed at base-line (AOR = 2.24, p= 0.010) | - Results only reported for the comparison of cross-sectional survey results between the 3 cities  - Base-line and end-line cross-sectional surveys did not necessarily include the same FSWs  - No explanation/description of the characteristics that OR were adjusted for  - Significant results about cervical screening uptake were only found in Durban (South Africa)  - No report of the results of the focus group discussions  - The sampling method used increases the risk of reporting (social desirability), classification, and selection bias  **Ethical consideration**:  - Written informed consent given by all study participants  - Use of non-identifying survey codes + storing information in password-protected computers ensured confidentiality  **Funding**:  - Funds received from the European Union Seventh Framework Programme  - In Mozambique, the International Department Flanders gave additional funding | - There was an increase of SRH service utilization (including CC screening) by FSW in Durban thanks to a higher use of both FSW targeted services and public health facilities  - The introduction (in Durban) in public health facilities of “navigators” that were peer outreach workers, and had the role to :   - Act as an interface between FSWs visiting the facility and the healthcare providers - Provide information regarding SHR services offered - Track referred patients   might have play a role in the results observed. |

| ***“Cervical cancer screening and treatment of cervical intraepithelial neoplasia in female sex workers using “screen and treat” approach” (3).*** Smita Joshi, Vinay Kulkarni, Trupti Darak, Uma Mahajan, Yogesh Srivastava, Sanjay Gupta, Sumitra Krishnan, Mahesh Mandolkar, Alok Chandra Bharti. (2015) | | | | | |
| --- | --- | --- | --- | --- | --- |
| Study objective  Study design  Country/Year of study | Sampling  Intervention | Methodology | Main results | Limitations  Ethical consideration  Funding sources | Conclusion/ Recommandations |
| **Objective**:  • To evaluate the feasibility of a Screen and Treat approach for CC prevention  • To evaluate the performance of different screening tests (cytology, VIA, visual inspection with Lugol’s iodine (VILI)) among FSWs  **Design**: Cross-sectional  **Country**: India / October 2012- February 2013 | **Sampling**: Convenience sampling (referral of FSWs to the designated study clinic by NGOs’ counselors and peer educators)  n=300  **Intervention**:  Screen and treat approach | 1. Collection of data (participants’ characteristics) through a structured questionnaire  2. Collection of venous blood for STI screening (HIV, Hepatitis B, syphilis, and CD4 + HIV viral load for HIV-infected FSWs)  3. “Appropriate post-test counseling was done to participants detected with HIV infection” (3), and treatment for STI was provided if needed  4. A trained nurse did a speculum examination + cervical specimen collection for cytology + cervical smear to FSWs  5. Two trained nurses in visual screening methods did a cervical screening using VIA  6. A trained doctor did the colposcopy, and the application of Lugol’s iodine was part of the colposcopic evaluation  7. If there was a lesion suggestive of any colposcopic abnormality, findings were explained to the woman and multiple punch biopsies were collected from the abnormal area for histopathology  8. Women having lesions with no suspicion of invasive cancer were treated immediately using cold coagulation  9. All women’s cervical biopsies “were analyzed and the results were reported by two pathologists using CIN terminology […] and then a consensus diagnosis was reached” (3)  10. Data were analyzed using STATA 12.0 | - VIA was positive in 15.2%, VILI in 12.2% and cytology at atypical squamous cells of undetermined significance threshold was positive in 22.7% women  - Mild dysplasia (CIN 1) was detected for 3.4% of women  - Moderate dysplasia (CIN 2) was detected for 1.7% of women  - Severe dysplasia (CIN 3) was detected for 3.1% of women  - All women with CIN 2-3 were HIV infected  - 9.12% underwent cervical punch biopsies and cold coagulation treatment (all FSWs, except 1, accepted biopsies and treatment during the same screening visit) | - Limitations of the study are not clearly stated by the authors  - Non-probabilistic sampling method  - Small sample size  **Ethical consideration**:  - Project approved by the ethics committee  - Each participant gave a written informed consent  **Funding**: Not specified | - “Prevention of cervical cancer among FSWs has remained neglected” (3)  - The Screen and Treat approach allows for the early screening, detection, and treatment of cervical abnormalities among a highly mobile population where follow-up visits are hard to achieve  - This approach is feasible and allows FSWs to also access CC healthcare  - FSWs well accepted the program (including screening and treatment), and organizations providing care and support to FSWs could include CC screening in their program by this mean.  - “Cervical cancer screening and treatment of CIN using “screen and treat” strategy needs to be integrated into targeted interventions that are ongoing for prevention of HIV infection and care and support of HIV-infected FSWs.” (3) |

| ***“Silent killer of the night: a feasibility study of an outreach well-women clinic for cervical cancer screening in female sex workers in Hong Kong” (4).*** *W.C.W. WONG, Y.T. WUN, K.W. CHAN & Y. LIU* (2008) | | | | | |
| --- | --- | --- | --- | --- | --- |
| Study objective  Study design  Country/Year of study | Sampling  Intervention | Methodology | Main results | Limitations  Ethical consideration  Funding sources | Conclusion/ Recommendations |
| **Objective**: To see if an outreach model could be a feasible option for early detections of preinvasive CC in women that are both FSWs and illegal migrant workers  **Design**: Cross-sectional  **Country**: Hong-Kong / January 2005 – December 2005 | **Sampling:** : Convenience sampling (outreach workers from Ziteng invited FSW to the clinic + snowball)  n=245 (Underwent Pap Smear n= 236)  **Intervention**: Start of an outreach well-women clinic (at the Ziteng NGO), twice monthly, for FSWs where Pap smears were provided (in addition to other health services) | 1. FSWs filled in a questionnaire regarding lifestyles & demographic information  2. Conduction of gynecological history, physical and gynecological examination (with cervical smear using liquid-based Pap technique) by a volunteer doctor, and relevant examinations addressing an individual’s concern.  3. Health education was given during the session, and referrals were arranged, if appropriate.  4. Samples were sent to a private accredited laboratory (results were reported in CIN)  5. Results were explained to FSWs during a follow-up visit 1-4 weeks later. If the woman could not attend the follow-up visit, the doctor explained the results by telephone.  6. If results indicated CIN 2 or 3, a referral letter was given to the woman to attend a gynecologist of choice  7. For abnormal smear results, 2 options were offered :  • Repeating another smear in 3-6 months  • To be referred to a specialist  8. Data were managed and analyzed using SPSS version 13.0 | - 64.5% of all women never had a cervical smear  - 87.7% of women who underwent Pap smear had “normal” results  - 11.9% of women who underwent Pap smear were lost to follow-up (so they couldn’t receive their results)  - 2.5%, 6.8%, and 3% of women had reactive changes in squamous cells, CIN I and CIN 2-3 results, respectively  - Among all the women detected with abnormal Pap smears, only 10% have had a previous cervical smear  - 44.8% of women with abnormal Pap results (reactive changes and CIN 1) could not be contacted. Among the ones that could be contacted, 9 out of 16 were referred for further management | - Non-probabilistic sampling method  - Sample size was limited due to limited resources that allowed the researcher to offer only 2 clinical sessions a month  - Unavailability of non-response rate and characteristics of the non-respondents  - No data collection about how many FSWs returned for a repeated cervical smear at 1 year  - High rates of women with abnormal results lost to follow-up  **Ethical consideration**:  Not specified  **Funding**: Not specified | - Pre-invasive lesions were higher in FSWs than in the general population  - Such an outreach clinic is feasible, effective in identifying abnormal early changes in cervical cells as well as ensuring referral for further management and care, and valuable in preventing CC in FSW  - Necessity to accommodate opening hours to meet FSWs SRH needs |

| ***“Can Human Papillomavirus DNA Self-sampling be an Acceptable and Reliable Option for Cervical Cancer Screening in Female Sex Workers?” (5).*** *Eliza L.Y Wong, Annie W.L. Cheung, Fenwei Huang, Josette S.Y. Chor* (2018) | | | | | |
| --- | --- | --- | --- | --- | --- |
| Study objective  Study design  Country/Year of study | Sampling  Intervention | Methodology | Main results | Limitations  Ethical consideration  Funding sources | Conclusion/ Recommendations |
| **Objective**: To explore the acceptability and reliability of HPV DNA self-sampling as an alternative option for CC screening among FSWs  **Design**: Clinical trial  **Country**: Hong Kong / November 2009 and May 2010 | **Sampling**: Convenience sampling (participants were recruited from an NGO that provides health services to FSWs)  n=68  **Intervention**:  • Interviews using a structured questionnaire (3 sections: attitudes, behavioral aspects, sociodemographic information)  • HPV-DNA self-sampling  • Clinician-collected HPV DNA testing and Pap test | 1. Participants carried out HPV DNA self-sampling  2. First interview where “participants were invited to a face-to-face individual interview with a research officer using a structured questionnaire to explore the degree of acceptability (attitudes and preference) of HPV DNA self-sampling versus clinician sampling” (5)  3. About 20-30min after the self-sampling, participants underwent clinician sampling (Pap-test n=65 & clinician HPV DNA testing n=68)  4. Second interview (same circumstances and objectives than the first one)  All cervical screening tests were analyzed in the university laboratory for 15 high-risk HPV types and 21 low-risk HPV types  “The questionnaire was derived from an extensive literature review” (5), the Cronbach’s α coefficient was calculated for the 1^st^ section (= attitudes), and finally, the questionnaire was pre-tested with 10 women.  Attitudes toward HPV-DNA self-sampling VS clinician sampling were evaluated through 7 dimensions (embarrassment, discomfort, anxiety, unpleasantness, degree of scare and relaxation, confidence in the test being performed correctly) using a 5-point rating scale  Data were managed and analyzed using PASW version 18. | - 76.2% of women already had undergone a Pap test in their life  - “65.6% of participants indicated a preference for adopting HPV DNA self-sampling in the future or accepting both methods because it was convenient, simple, and less frightening” (5)  - 34.4% preferred clinician sampling as they were more confident in the procedure carried out by healthcare professionals  - 5.9% didn’t have plans or opinions on preferences for future cervical screening  - 86.7% of women with no history of Pap test had a preference for using the self-sampling method, either at home or a clinic, in the future against 58.3% of the women with a Pap test history.  - 1.5%, 3.1%, and 9.2% of women who underwent Pap test had a high-grade squamous intraepithelial lesion, low-grade squamous intraepithelial lesion, and ASCUS results, respectively.  - “Participants felt less embarrassed, anxious, unpleasantness, and frightened and more relaxed with the self-sampling method” (5)  - 70.6% of women were confident that self-sampling was performed correctly, and this percentage raised to 91.2% regarding the clinician-sampling method  - One of the greatest obstacles to Pap test was embarrassment  - HPV DNA testing of clinician-obtained samples had greater sensitivity and specificity than self-collected samples  - 10 HPV-DNA discordant results (between self and clinician sampling) were found :  • 6 positive results in self-collected samples were negative in  clinician-collected ones  • 4 negative results in self-collected samples were positive in  clinician-collected ones | - Limitations of the study are not clearly stated by the authors  - Non-probabilistic sampling method  - Small sample-size  - Characteristics of the non-respondents to the sociodemographic questionnaires and the ones who refused the Pap test are not given  **Ethical consideration**:  Each participant gave a consent and had a study identification number  **Funding**:  By Direct Grant of Research, Chinese University of Hong-Kong | - HPV-DNA self-sampling method “is feasible and an easy-to-implement option that could potentially be integrated into conventional cervical screening to increase its uptake rate” (5) to reduce the morbidity and mortality of CC  - High acceptance of the use of self-sampling as a screening tool in the future  - HPV-DNA self-sampling could be used to improve the response rate & compliance to cervical screening in non-attenders (such as FSWs)  - Access to self-sampling at NGOs providing services to FSWs could improve screening rates among this population. |

| ***“Cervical cancer screening in rural South Africa among HIV-infected migrant farm workers and sex workers” (6).*** *Omara Afzal, Molly Lieber, Peter Dottino, Ann Marie Beddoe* (2017) | | | | | |
| --- | --- | --- | --- | --- | --- |
| Study objective  Study design  Country/Year of study | Sampling  Intervention | Methodology | Main results | Limitations  Ethical consideration  Funding sources | Conclusion/ Recommendations |
| **Objective**:  • To integrate the See and Treat approach into current HIV care offered by local providers  • To obtain prevalence data on VIA positivity  • To evaluate efficacy of the program  • To collect demographic and risk factor data for use in future educational and interventions programs in the region  **Design**: Cross-sectional study  **Country**: South-Africa (Limpopo province) / 2015 | **Sampling**: Purposive sampling  n=403 (n=97 counting FSWs only)  **Intervention**: See and Treat approach | 1. Forging of a collaboration with the Hoedspruit Training Trust (HTT) NGO, which created the Hlokomela Clinic (to improve the migrants health and permanent farm workers through service provision)  2. “Female patients attending the clinic for HIV counseling, treatment or follow-up were offered cervical cancer screening as part of their routine HIV care” (6)  3. Participants completed questionnaires with trained health workers  4. Then, participants were screened, by beforehand trained nurses, using VIA  5. If the VIA examination was positive, participants were offered cryotherapy for treatment (also done by the trained nurses)  6. “Clinic charts were reviewed retrospectively for Pap smear results from the previous year” (6)  7. “Prevalence data was collected on VIA results and cryotherapy” (6)  8. At 12 and 18 months post-initial screening, follow-up programs were performed  9. “Clinic charts from the initial cohort of patients screened were reviewed to collect data on recent Pap smear results and one-year post-screening VIA results” (6)  Data were managed and analyzed using SPSS.  5 local nurses underwent training :  • A see-and-treat workshop: 3-month supervisory period during which they performed 50 supervised VIA and 15 supervised cryotherapy procedures  • 1-year post-program implementation: refresher training where they were supervised and reassessed in quality and performance of conducting VIA | - 32.9% of FSWs were HIV positive  - 27.8% of FSWs had a positive VIA compared to 31.7% of farm workers (all of the latter were HIV positive)  - 91.6% of women who had a positive VIA underwent cryotherapy (6 patients with large lesions were referred to the hospital for cone biopsy)  - At 1-year post-program implementation, clinic records showed that 193 additional patients had undergone VIA: 41.4% were VIA positive and 35.2% of them received treatment with cryotherapy  - Women who had initially screened VIA positive and underwent repeat VIA after 1 year were essentially now VIA negative (4 patients remained VIA positive and 2 underwent 2^nd^ cryotherapy, and 2 were referred for further evaluation and biopsy).  - “Patients with examinations consistent with invasive carcinoma were often lost to follow-up without ever being evaluated” (6)  - More than 50% of participants were lost to follow-up after the initial screening program | - Non-probabilistic sampling method  - Risk of selection bias  - High rates of loss to follow-up  - No disaggregated data between FSWs and farm workers regarding:  • Women who  had VIA  positive results  and had a  cryotherapy  • Women who  came back 18  months after  the program  implementation  **Ethical consideration**:  Not specified  **Funding**: From thewomen.org | - Even if data were aggregated, the study showed a successful integration of CC screening using VIA into an existing HIV treatment and prevention clinic  - Thanks to the See and Treat approach, “abnormal results could be readily addressed and treated promptly” (6)  - See and Treat approach allow to target hard to reach and highly-moving population  - The See and Treat approach is feasible and can improve HIV clinics’ screening methods  - Education, training, and supervision helped create community ownership of healthcare which contributed to the project’s efficacy and sustainability  - Difficulty in referral for further testing and cancer care (As soon as further investigations are needed, women’s compliance with CC screening and treatment becomes challenging)  - Lost to follow-up after the initial screening program is widespread  - “Improved infrastructure and referral process [for high-risk populations (including FSWs)] is a highly unaddressed need” (6) |

| ***“Cervical Cancer Screening in HIV-Positive Farmers in South Africa: Mixed-Method Assessment” (7)*** *Molly Lieber, Omara Afzal, Kathryn Shaia, Adrienne Mandelberger, Christine Du Preez and Ann Marie Beddoe* (2019) | | | | | |
| --- | --- | --- | --- | --- | --- |
| Study objective  Study design  Country/Year of study | Sampling  Intervention | Methodology | Main results | Limitations  Ethical consideration  Funding sources | Conclusion/ Recommendations |
| **Objective:**  • To determine the quality and sustainability of the implemented program 18 months post-implementation (See and Treat approach for migrant farm workers and sex workers)  • To determine the next steps to improve the quality of the program in the future  **Design:** Mixed-method assessment  **Country:** South Africa (Limpopo province) / 2016 | **Sampling**: Convenience sampling (health providers, ancillary health workers, and patients recruited from the clinic)  Interviews:  n=18 (12 patients, 3 counselors, 1 nurse, 2 peer educators)  *Quantitative data: n*=403  **Intervention**:  • Interviews  • 1 focus group with nurses  • Clinical observation  • Review of charts  • Review of clinic logs | **Qualitative data:**  1. Audio recorded interviews were completed using a 12 questions guide regarding the acceptability of CC screening (particularly VIA) among health workers and patients, but also on understanding of screening, knowledge of CC and HPV, and barriers to receiving reproductive healthcare  2. Conduction of 1 focus group “with nurses to assess their perspectives and experiences with the addition of cervical cancer screening into their routine HIV counseling and treatment program” (7)  3. Observation of health workers who underwent initial training in CC screening by the ObGyn team from the USA  4. Observation of providers during patient interviews, speculum examinations, and VIA performance. They were then graded as “poor/fair/good.”  Nurses and community health workers acted as interpreters for patients whose 1^st^ language was not English.  Interviews were transcribed and coded to elicit themes by research team members.  **Quantitative data**: Pap smear, VIA results, and cryotherapy were obtained from chart reviews and patient logs.  5. Review of charts of the 1^st^ patient cohort and those screened at program rollout  6. 18 months post-initial program integration logs were evaluated | Interviews with patients:  - Patients felt more at ease (regarding the position required for undergoing screening, the tool used, and the results of the exam) with explanations from the nurses at the clinic  - Good understanding, by patients, of the purpose of Pap smears and VIA screening, and of the importance of screening to detect lesions at early stages.  - Patients satisfied with the method of education used (train the trainer)  - Community educators walking door to door to increase awareness was seen as valuable  - Expansion of the CC screening program to other clinics and more education were the gaps that needed to be filled according to patients  - Patients expressed a need for more information about cancer (causes, symptoms, stages, available treatment options)  Focus group with nurses:  - High levels of understanding and awareness of :  • CC screening  • Privacy concerns  • Negative perceptions of medical care  Both of the latter being acknowledged as barriers to screening  - Sense of empowerment for the acquired skills  - Expressed the need for continuous education to better face challenges they were unfamiliar with  Observations: Healthcare workers who received education on performing VIA and cryotherapy maintained and continued these skills.  Chart review :  - Very high percentage of patients lost to follow-up (60%)  - Most clinic records were incomplete or missing | - Non-probabilistic sampling method  - Small sample size  - No disaggregated data regarding sex workers  - High rates of loss to follow-up  - Lack of consistency and accuracy in record keeping (possible bias related to unreliability of content)  - Translated interviews may have been subject to bias or limited patient response given the provider-patient relationship  **Ethical consideration**:  - Institutional review board approval obtained  **Funding**: From thewomen.org | - See and Treat approach increased awareness of CC among women  - Such approach can be successfully implemented in already existing STI clinic  - Program was still ongoing (even if facing a shortage of staff)  - Even if there was a high rate of loss to follow-up, the program has been successful as it was still ongoing after the study and allowed to increase awareness of CC by patients & healthcare providers  - Program sustainability was challenging to assess as many patients were lost to follow-up, but healthcare workers are continuing to perform See and Treat exams, and awareness of CC is increasing, so program sustainability was partly achieved in this way |

| ***“Cervical cancer screening among marginalized women: A cross-sectional intervention study” (8)*** Thomas HG Bongaerts, Marlieke Ridder, Josephina CJ Vermeer-*Mens Jeanette J Plukkel, Mattijs E Numans, Frederike L Büchner* (2021) | | | | | |
| --- | --- | --- | --- | --- | --- |
| Study objective  Study design  Country/Year of study | Sampling  Intervention | Methodology | Main results | Limitations  Ethical consideration  Funding sources | Conclusion/ Recommendations |
| **Objective:**  • To identify the prevalence of (pre)cancerous abnormalities among marginalized women (referring to sex workers, homeless women, uninsured women, and undocumented women)  • “To explore invitation approaches to enhance the screening uptake among this specific group of women” (8)  **Design:** Cross-sectionnal  **Country:** Netherlands (Rotterdam) / February-May 2019 | **Sampling**: Purposive sampling  “Recruitment of the women took place at homeless shelters, day and night shelters for undocumented people, respite care locations, safe houses for sexual trafficking victims, **in** brothels, and sex worker walk-in houses” (8)  n=74 (FSWs n=20)  **Intervention**:  • Direct and indirect invitations for CC screening  • Liquid-based cytology sample | 1. Either a **direct** or **indirect** invitation approach was used for recruiting the women :   - Direct invitation “was done during the consultation hours of the street doctor or combined with the consultations for STIs by sexual health workers” (8). It consisted of a **proactive offer of an immediate cervical smear** - Indirect invitations “consisted of distributing posters in relevant areas and announcements on a website, with information about the opportunity to have a cervical smear performed” (8)   2. “Mails were sent to all known care providers or case managers of the population under study, with the option to make an appointment for their client to have a cervical smear” (8)  3. Women were screened directly at the locations where they would already be present to work, reside or receive care (Liquid-based cytology sample was the screening method used)  4. “Participants were informed of their test results by means of consultations, text messages, and phone calls; usually directly to the participant but occasionally to their care providers” (8)  5. Referral to a gynecologist was done by the street doctor (or own GP)  A female street doctor and a female nurse familiar with the study population were performing the cervical smears  All data were managed and analyzed using IBM SPSS Statistics 25 | - 35% of women had a positive result to the High-Risk HPV test (HR-HPV), and 20% had abnormal smears.  - In total, 16% of women had a positive result to HR-HPV test and an abnormal smear  - 92% of women were recruited via the direct invitation approach. Among the women recruited via the indirect approach, it was through appointments made by their care providers for 83% of them.  - The public health safety-net team was essential to mitigate the loss to follow-up | - Non-probabilistic sampling method  - Small sample-size  - A direct comparison of invitation methods was not possible because not all the approaches were equally suitable at every location  - Unavailability of non-response rate and characteristics of the non-respondents  - No data on HR-HPV vaccination status, and it can influence the study results (especially for the age group < 21yo)  - Unavailability of disaggregated data  - The major drawback of the tailor-made approach is that it is time-consuming and it depends on the availability of a network and the setting  **Ethical consideration**:  - Women gave consent to share their medical record for research  - Data were anonymized  - Study approved by the Ethics Committee  **Funding**: Stichting Bevolkingsoonderzoek Zuid-West covered part of the costs of the research | - National population-based cancer screening programs for cervical cancer are missing out on marginalized populations, such as FSWs  - Due to lack of permanent address or being undocumented, marginalized women often fail to receive invitation letters sent by the regionally coordinated national cancer screening program or are not invited at all. Therefore, new invitation approaches are needed.  - A direct proactive approach is a very effective way to stimulate screening participation among marginalized women (including FSWs)  - Being proactive and making use of close care providers seems crucial to address the health needs of this specific population  - Peer influence is invaluable (e.g., “several participants became so convinced of the importance of screening they encouraged other women to participate in cervical cancer screening” (8))  - Engagement and participation based on trust is crucial (an approach based on creating a safe environment seemed effective)  “Recommendations for implementing a cervical screening program for marginalized women:  1. Be proactive as a care provider 2. Provide the cervical smear at the locations where the women work, reside, or receive care 3. Use a trusted care provider on the location for recruitment and the introduction of the program 4. Use female medical teams 5. Involve peers (give them a role in educating and raising awareness) 6. Consider screening from a younger age onward, starting at the age of 25 is recommended 7. Make sure follow-up is guaranteed and explore regionally which organizations can cooperate” (8). |

| ***“Experiences of a ‘screen and treat’ cervical cancer prevention programme among brothel-based female sex workers in Bangladesh: A qualitative interview study” (9)*** *Emma Wilson, Sharmani Barnard , Samiya Mahmood, Olivia Nuccio, Sujit D Rathod, Raveena Chowdhury, Sabitri Sapkota, Tanzila Tabassum, Shah Halimur Rashid and Catherine Verde Hashim* (2021) | | | | | |
| --- | --- | --- | --- | --- | --- |
| Study objective  Study design  Country/Year of study | Sampling  Intervention | Methodology | Main results (regarding cervical screening and cryotherapy only) | Limitations  Ethical consideration  Funding sources | Conclusion/ Recommendations |
| **Objective:**  • To understand sex workers perspectives of Screen and Treat programs  • To gain insights about FSWs management of the post-treatment healing phase (WHO post-treatment guidance are to abstain from sex or use condoms consistently for 4 weeks)  • “To understand SWs contraceptive preferences and their use of menstrual regulation [⬄ abortion] services to improve delivery of SRH services to this population” (9)  **Design:** Qualitative  **Country:** Bangladesh / October-November 2018 | **Sampling**: Purposive sampling  Brothel-based FSWs (n=16) and brothel leaders (who had also worked as SWs) (n=6) who received cryotherapy from the “Marie Stopes Bangladesh” (MSB) Screen and Treat program.  Total : n=22  **Intervention**: None | 1. Two trained female research assistants (that had previously worked for the MSB project) carried out semi-structured, audio-recorded interviews, in Bengali, in a private space within the brothel complex  2. Research assistants recorded field notes during and after each interview  3. Interviews were transcribed verbatim  4. Transcripts were translated into English by 2 professional translators  5. A thematic content analysis was conducted by 2 researchers using deductive and inductive data-driven coding  6. Data were coded and managed using Microsoft Excel  The topic guide for interviews was pre-tested with 2 FSWs before the initiation of data collection.  Topics discussed during interviews were :  • Health and relationships  • Experiences of the MSB screen and treat programs  • Understanding and adherence to post-treatment guidance  • Contraceptive preferences  • Menstrual regulation-seeking behaviors  Research assistants :  • Had prior experience of conducting qualitative research  • Received 3 days of training on qualitative interviewing techniques, study procedures, and research ethics  A research manager oversaw data collection. | - Key motivating factors for attending the MSB Screen and Treat program were :  • Opportunity to learn their cancer  status  • Receive a free treatment  • Potentially resolve ongoing  symptoms (discharge, vaginal  itching, abdominal pain)  - Many FSWs felt frightened of the medical equipment  - Some FSWs understood that the screening would involve a hysterectomy  - Cryotherapy was painful for the participants  - Most SWs felt reassured by staff and had high trust in MSB personnel. The latter were respectful and showed “affection” to the participant  - Most SWs had difficulty in following the post-treatment guidelines, and most of them re-engaged in sex work few days after cryotherapy (sex work is a necessity for daily survival).  - One brothel leader refused that a FSW attend the CC screening program to avoid loss of earning  - Brothel “leaders feared that MSB staff might report them to the authorities” (9) | - Risk of recall bias (interviews were conducted 12-18 months after the participants underwent cryotherapy)  - Risk of response bias  - Limited generalizability and representativity of the results  - Uncertainty about data saturation regarding adherence to post-treatment guidelines as only 2 FSWs complied with them  **Ethical consideration**:  - All participants provided written informed consent  - Ethical approval was obtained from the Ethics review committee + Bangladesh Medical Research Council  **Funding**: Bill & Melinda Gates Foundation | - “Service innovations are required to increase the acceptability of cervical cancer screening for SWs and address their broader sexual and reproductive health needs” (9).  - Screen and treat approach has been seen as valuable, but following the post-treatment guidelines seems nearly impossible as FSWs are dependent on their activity if they want to meet their needs  - Sensibilisation beforehand of screening procedures is needed to make sure the latter is well understood  - The MSB Screen and Treat program has ceased due to a lack of funding before this research was conducted |

| ***“Medical health care for Viennese prostitutes”(10)*** *Stary A., Kopp W., Söltz-szöts J.* (1989) | | | | | |
| --- | --- | --- | --- | --- | --- |
| Study objective  Study design  Country/Year of study | Sampling  Intervention | Methodology | Main results (regarding cervical screening only) | Limitations  Ethical consideration  Funding sources | Conclusion/ Recommendations |
| **Objective:**  • To see the evolution of STI and abnormal Pap smear prevalence between 1988 and 1989 among registered FSWs  • To compare STI and abnormal Pap smear prevalence between registered and non-registered FSWs  • To see the effectiveness of mandatory routine screening  **Design:** Cross-sectional  **Country:** Austria (Vienne) / (1989) | **Sampling**:  Convenience sampling  (Use of medical examination of sex workers who attend the medical examination in the STD clinic of the Public Health Office (PHO))  For Pap smears: n=991 in 1988 and n=958 in 1989  **Intervention**:  • Screening for main STDs (either weekly, every 6 weeks, or once a year)  • Medical exam (X-ray for TB, cutaneous inspection)  Pap smear was done once a year | 1. Realization of the weekly, every 6 weeks, and once-a-year medical examinations  2. Laboratory analysis and diagnosis of the different screened diseases  3. Analysis of the results  4. Comparison with previous years  “Since 1873, health surveillance has been conducted in Vienna for all prostitutes, and prostitution has been permitted only when investigations were routinely performed and recorded in a health book, which was carried by the prostitutes” (10). Registered sex workers have regular medical and laboratory examinations at the STI clinic of the PHO. Among non-registered sex workers, only a few of them were asked to attend the medical examination. | - 87% of scheduled weekly examinations among registered sex workers were completed  - Among registered FSW, 8.3% had an abnormal Pap test result in 1988, contrary to 6.8% in 1989  - Among registered FSW, 3.1% had high-risk lesions in 1988 against 1.6% in 1989 | **-** Authors do not expose the limitation of their research  - Non-probabilistic sampling method  - Methodology is not well described  - No information about what happened after being screened positive (either for STIs or cervical cancer)  **Ethical consideration**:  Not specified  **Funding**: Not specified | - Provided health service was well accepted by sex workers (high compliance to weekly medical examination)  - Registered sex workers have less positive results to STIs than the non-registered ones  - Prevalence of abnormal Pap smears was higher among sex workers than the general population  - Offering regular screening to FSWs :  • Gave the possibility for adequate therapy free of charge  • Improved FSWs' health education and understanding of preventive methods  - Improved access to health care services directly impact the prevalence of main STIs and abnormal Pap smear  - Decriminalization of prostitution enhance access to health care services, therefore better FSWs’ health and respect for human rights |

| ***“*** ***The sexual health of female sex workers compared with other women in England: analysis of cross-sectional data from genitourinary medicine clinics”(11)*** *Louise Mc Grath-Lone, Kimberly Marsh, Gwenda Hughes, Helen Ward (2013)* | | | | | |
| --- | --- | --- | --- | --- | --- |
| Study objective  Study design  Country/Year of study | Sampling  Intervention | Methodology | Main results | Limitations  Ethical consideration  Funding sources | Conclusion/ Recommendations |
| **Objective**: To compare the sexual health outcomes and service usage of FSWs with those of other females attending GUM Clinics in England  **Design**: Analysis of cross-sectional data  **Country**: England / 2011 | **Sampling**: All women recorded as SWs who visited the clinics between 1^st^ January and 31^st^ December 2011  (n= 2704)  **Intervention**:  None | 1. Extraction of GUMCAD (Genitourinary Medicine Clinic Activity Dataset) database containing all visits by females  2. Socio-demographic characteristics, the number of clinic visits, and use of the different services (i.e., receiving PEPSE, contraception, STI testing or cervical cytology in any visit of the year, HIV testing, and Hep B vaccination) variables were compared using Pearson χ2 tests.  3. “Univariate associations between SW status and diagnoses and demographic factors […] were investigated using logistic regression” (11)  4. “Factors with p-value < 0.10 were included in multivariate logistic regression” (11)  To compare UK-born and migrant FSWs, this analysis was repeated.  Stata V.12 was used for all analyses. | - SWs represented 0.4% of women who attended the GUM clinics in 2011  - FSWs made more visits than other female attendees (3.1 VS 1.7) and were more likely to have had a repeat visit  - FSWs mainly visited large clinics providing SW-specific services  - Non-testing services such as smear tests were more likely to be used by FSWs than other attendees  - FSWs are more likely to have abnormal cervical cell cytology (32.9% of FSWs against 16.3% of other female attendees had abnormal cervical cell cytology)  - 12.5% of FSWs used the smear test service available at the clinics (against 1.5% of other female attendees) | - The number of FSWs attending GUM clinics is likely to be underestimated (due to under-disclosure, lack of coding by staff, absence of guidelines on how SW status should be ascertained)  - FSW population attending GUM clinics might not be representative of the wider FSW population  - Generalizability of the results is limited  **Ethical consideration**:  Not specified  **Funding**: From the Wellcom Trust and the NIHR Imperial College Healthcare NHS Trust Biomedical Research Centre | - GUM clinics play a vital role in meeting FSWs’ broader SRH healthcare needs (as they use CC screening more than other attendees)  - Tailoring interventions and services for FSWs is necessary and will be useful (e.g., Location of outreach services, language offered to them)  - Sex-workers-specific services play a crucial role in FSWs’ sexual health care provision  - There are inequalities in terms of access to and awareness of suitable services for FSWs  - Still a lot of missed opportunities (1/8 FSWs had no sexual health screen, including for CC, despite being already engaged with healthcare services)  - Integrated approach needs to be adopted to improve the sexual and general health of FSWs  - “Overall, FSWs in England have access to high-quality sexual health care through the GUM clinic network” (11), but improvements still need to be made. |

| ***“*** ***Barriers to Follow-Up for Abnormal Papanicolaou Smears among Female Sex Workers in Lima, Peru” (12)*** *Devora Aharon, Martha Calderon, Vicky Solari, Patricia Alarcon, Joseph Zunt* (2017) | | | | | | | | | | |
| --- | --- | --- | --- | --- | --- | --- | --- | --- | --- | --- |
| Study objective  Study design  Country/Year of study | Sampling  Intervention | | Methodology | | Main results (regarding cervical screening and cryotherapy only) | | | Limitations  Ethical consideration  Funding sources | Conclusion/ Recommendations | |
| **Objective**: To identify barriers to follow-up for abnormal Pap smears among FSWs  **Design**: Mixed-method  **Country**: Peru / From March to July 2014 | **Sampling**: Convenient sampling (all FSWs attending the “Centro de Salud Alberto Barton del Callao” were invited to participate)  *Questionnaire :*  n=97  *Interviews*:  n=17 : 8 women who received follow-up care and 9 who did not  **Intervention**:  None | | 1. Questionnaire about :  - The patient’s history of receiving results for STI screened for at Barton  - Receival of follow-up care for the STI detected  - Reasons why they received follow-up  - Perceived barriers to not receiving follow-up  2. Reviews of medical records for all study participants  3. Guided in-depth interviews (addressing participant’s experience of having an abnormal Pap smear and the reasons for receiving or not follow-up care) for women who tested HPV positive or have had abnormal cytology in the past, conducted by a Peruvian psychologist (not a member of the clinic staff)  4. Analyses of the questionnaires using STATA and of interviews using NVivo software | | Quantitative results:  - 27.8% of women have had a history of abnormal Pap smear results, and 30% of them received follow-up treatment. 37% had not been informed of their screening results.  - 100% of women who tested positive for an STI received follow-up  Qualitative results: Most women had been screened between 1 and 5 months before the study  - 5/12 women who did not receive follow-up care had never been informed of the results of their Pap smear.  - 7/12 women knew the results but did not receive care  Then 3 women received follow-up, so in total, for interviews :   - n=8 for women who received follow-up - n=9 for women who did not   *Factors associated with lack of follow-up*:  • Not having been informed of abnormal results (due to lack of clinic’s procedures for recording when the test has been done, its result, and whether the latter has been transmitted to the patient)  • Lack of knowledge regarding CC, its causes, and screening possibilities  • Knowing someone who had a negative experience with follow-up for an abnormal Pap smear  • Being a migrant worker  *Factors associated with a successful follow-up :*  • In-depth knowledge of HPV, its relation with CC, screening, and treatment of cervical dysplasia  • Social support (family, friends, partner)  • Knowing someone who died of CC  *Factors not associated with obtaining follow-up care :*  • All women reported a high level of satisfaction with their providers and the medical system  • Decision to pursue or not follow-up care was attributed to fear in both group  • Women in both groups expressed the need to miss work days to be treated and recover as a challenge | | | - Limitations of the study are not clearly stated by the authors  - Limited validity of the reported satisfaction with providers and the medical system (because interviews have been conducted at the Barton clinic, a well-known and safe environment for FSWs)  - Limited generalizability to other FSWs population in other countries  **Ethical consideration**:  - Approved by the Institutional Review Board of the University of Washington  - Approved by the ethics committee  - Informed consent obtained orally (to protect the identities of study participants)  **Funding**: From the NIH Research Training Grant and the Arnhold Global Health Institute | - “Cervical cancer screening program alone is not sufficient to ensure women receive proper treatment” (12)  - Follow-up is more common among women who received and understood their test results, and the potential health consequences of those results  - Increased level of knowledge and understanding of the test results and disease process was associated with motivation to seek care  - Clinic providing more information on HPV infection and CC as well as additional counseling services was recommended by all women  - Delays between the test and the result + referral to a local hospital are likely to be associated with the disparity between rates of follow-up for HPV or other STI (not a possibility to have a same-day treatment)  - 2 interventions have been implemented after the study:  • Creation of standardized forms for recording Pap smear exam dates, results, and follow-up care  • Creation of a brochure on Pap smears, HPV, and CC to be distributed to FSWs during their visits | |
| ***Visual inspection with acetic acid (VIA) positivity among female sex workers: a cross-sectional study highlighting one-year experiences in early detection of pre-cancerous and cancerous cervical lesions in Kampala, Uganda” (13)*** *Gertrude Namale, Yunia Mayanja, Onesmus Kamacooko, Daniel Bagiire, Agnes Ssali, Janet Seeley, Robert Newton and Anatoli Kamali* (2021) | | | | | | | | | | |
| Study objective  Study design  Country/Year of study | | Sampling  Intervention | | Methodology | | Main results | Limitations  Ethical consideration  Funding sources | | | Conclusion/ Recommendations |
| **Objective**: “To investigate VIA positivity among FSWs in the early detection of pre-cancerous and cancerous cervical lesions” (13)  **Design**: Cross-sectional  **Country**: Uganda (Kampala) / From June 2014 to July 2015 | | **Sampling**: Convenient sampling (FSWs who were attending their 3-monthly routine Good Health for Women Project (GHWP) clinic visits and interested/eligible for VIA screening)  n= 719  **Intervention**:  • Screen and Treat approach  • STI screening and treatment | | 1. Before the implementation of the program, 5-days training to nurses and clinicians for VIA procedures, then 2 weeks of supervised practice.  2. To improve quality, the staff underwent periodic reorientation sessions and routine quality checks.  3. FSWs benefited from awareness campaigns by the trained staff during health education activities at the clinic.  4. Enrolled women participated to a face-to-face interview and counsellors collected socio-demographic, sexual behavior and clinical characteristics data.  5. Women first underwent routine vaginal examination and then VIA procedure  6. Results were obtained and classified as negative, positive or inconclusive, 1-3min after the procedure was performed.  7. If there was uncertainty about the results, women were recalled for rescreening  8. All positive results were immediately reported to women and they were offered accompanied referral to the senior gynecologist (in a tertiary hospital) for colposcopy and further management on the same day (Screen and Treat approach).  9. After the VIA procedure, all women had a pregnancy test, and a general physical and gynecological exam  Only for positive women : All procedures were explained to the woman    10. VIA positive women underwent colposcopy at the tertiary hospital to grade the lesions, which were reported as normal, inflammation, probable low or high-grade precancerous lesions, suspected invasive cancer. Cervical biopsy were analyzed and results reported using CIN system.  11. Depending on the results, immediate cryotherapy treatment or loop electrosurgical excision procedure (LEEP) was proposed to eligible women. Women who had invasive cervical cancer were managed following Uganda’s guidelines.  12. Results were explained to the participants during their 1-4 weeks later follow-up visit  13. All treated women received a one-week antibiotic therapy.  14. Women who were diagnosed with an STI were offered free treatment at the GHWP clinic, and HIV positive women were enrolled into care.  15. Women were “encouraged to invite their male regular partner to the clinic for free HIV/STIs screening and treatment as well as counselling on safe medical male circumcision and condom use” (13).  To ensure all women with positive results receive treatment, all procedures done at the referral hospital were paid by the project.  All data were analyzed using STATA 14.0. | | - 6% of women were VIA positive and 65% of them were referred for colposcopy and 35% were lost to follow-up  - 26 women had a colposcopy:  • 2 had inflammation  • 13 had low-grade  precancerous lesions  • 7 had high-grade  lesions  • 4 had a suspected  invasive cancer  - The 2 women who had inflammation were treated and did not undergo biopsy  - 24 biopsy results :  • 6 had normal results  or inflammation  • 1 had CIN1  • 13 had CIN2/3  • 4 had invasive cancer  - 11/14 with CIN1 & CIN2/3 had accepted immediate treatment :  • 8 cryotherapy  • 3 LEEP  - All women with invasive cancer complied with treatment | - Non-probabilistic sampling method  - High rates of loss to follow-up  - Generalizability of results is limited (due to the fact that it’s a single-site study)  - Misclassification bias : Limited experience and level of skill by the nurses and clinicians regarding VIA procedures and results interpretation + tendency to label unclear VIA findings as negative 🡪 leading to lower rates of VIA positivity than expected (and observed in other settings) in this high-risk population  - VIA is not a very reproducible test  - No investigation of HPV subtypes prevalence  - The project had a lot of financial limitations, which prevent from the development of other interventions  **Ethical consideration**:  - Written informed consent obtained from all participants for qualitative and quantitative data separately  - All participants were anonymized by an identification number  - Approved by the Ethics Committee, the Uganda Virus Research Institute and the Uganda National Council for Science and Technology  **Funding**: From the UK Medical Research Council and the UK Department for International Development | | | - FSWs have a high prevalence of CIN 2/3 highlighting the necessity to improve CC screening program for this high-risk population  - Integration of CC screening into routine HIV care can help in reaching the most at-risk women  - The Screen and Treat approach is very suitable for FSWs, as they are a highly mobile population, increasing the risk of loss to follow-up  - Screen and Treat strategies need to be integrated into other FSWs targeted interventions (e.g. HIV services)  - Necessity to develop collaboration with NGOs or FSW-led organizations to set up accessible health services that integrate CC screening  - Necessity to improve CC screening interventions for FSWs to reduce the cancer burden and mortality among this population. |

**Supplementary Table 1.** Summary of the 13 included studies.

**2. References**

1. Reza-Paul S, Lazarus L, Maiya R, Venukumar KT, Lakshmi B, Roy A, et al. Delivering community-led integrated HIV and sexual and reproductive health services for sex workers: A mixed methods evaluation of the DIFFER study in Mysore, South India. PloS One. 2019;14(6):e0218654.

2. Lafort Y, Greener L, Lessitala F, Chabeda S, Greener R, Beksinska M, et al. Effect of a ‘diagonal’ intervention on uptake of HIV and reproductive health services by female sex workers in three sub-Saharan African cities. Trop Med Int Health. 2018;23(7):774–84.

3. Joshi S, Kulkarni V, Darak T, Mahajan U, Srivastava Y, Gupta S, et al. Cervical cancer screening and treatment of cervical intraepithelial neoplasia in female sex workers using “screen and treat” approach. Int J Womens Health. 2015 May 4;7:477–83.

4. Wong WCW, Wun YT, Chan KW, Liu Y. Silent killer of the night: a feasibility study of an outreach well-women clinic for cervical cancer screening in female sex workers in Hong Kong. Int J Gynecol Cancer Off J Int Gynecol Cancer Soc. 2008 Feb;18(1):110–5.

5. Wong ELY, Cheung AWL, Huang F, Chor JSY. Can Human Papillomavirus DNA Self-sampling be an Acceptable and Reliable Option for Cervical Cancer Screening in Female Sex Workers? Cancer Nurs. 2018 Feb;41(1):45–52.

6. Afzal O, Lieber M, Dottino P, Beddoe AM. Cervical cancer screening in rural South Africa among HIV-infected migrant farm workers and sex workers. Gynecol Oncol Rep. 2017 May;20:18–21.

7. Lieber M, Afzal O, Shaia K, Mandelberger A, Preez CD, Beddoe AM. Cervical Cancer Screening in HIV-Positive Farmers in South Africa: Mixed-Method Assessment. Ann Glob Health. 2019 Apr 15;85(1):58.

8. Bongaerts THG, Ridder M, Vermeer-Mens JCJ, Plukkel JJ, Numans ME, Büchner FL. Cervical Cancer Screening Among Marginalized Women: A Cross-Sectional Intervention Study. Int J Womens Health. 2021 Jun 8;13:549–56.

9. Wilson E, Barnard S, Mahmood S, Nuccio O, Rathod SD, Chowdhury R, et al. Experiences of a ‘screen and treat’ cervical cancer prevention programme among brothel-based female sex workers in Bangladesh: A qualitative interview study. Womens Health. 2021 Sep 24;17:17455065211047772.

10. Stary A, Kopp W, Söltz-Szöts J. Medical health care for Viennese prostitutes. Sex Transm Dis. 1991 Sep;18(3):159–65.

11. Mc Grath-Lone L, Marsh K, Hughes G, Ward H. The sexual health of female sex workers compared with other women in England: analysis of cross-sectional data from genitourinary medicine clinics. Sex Transm Infect. 2014 Jun;90(4):344–50.

12. Aharon D, Calderon M, Solari V, Alarcon P, Zunt J. Barriers to Follow-Up for Abnormal Papanicolaou Smears among Female Sex Workers in Lima, Peru. PLoS ONE. 2017 Jan 6;12(1):e0169327.

13. Namale G, Mayanja Y, Kamacooko O, Bagiire D, Ssali A, Seeley J, et al. Visual inspection with acetic acid (VIA) positivity among female sex workers: a cross-sectional study highlighting one-year experiences in early detection of pre-cancerous and cancerous cervical lesions in Kampala, Uganda. Infect Agent Cancer. 2021 May 11;16:31.
